# Rhythmicity and waves in the cortex of single cells

**DOI:** 10.1098/rstb.2017.0116

**Published:** 2018-04-09

**Authors:** Yang Yang, Min Wu

**Affiliations:** Department of Biological Sciences, Centre for Bioimaging Sciences, Mechanobiology Institute, National University of Singapore, Singapore

**Keywords:** cortical oscillations, actin waves, single-cell pattern formation, activator–inhibitor, excitability, network biology

## Abstract

Emergence of dynamic patterns in the form of oscillations and waves on the cortex of single cells is a fascinating and enigmatic phenomenon. Here we outline various theoretical frameworks used to model pattern formation with the goal of reducing complex, heterogeneous patterns into key parameters that are biologically tractable. We also review progress made in recent years on the quantitative and molecular definitions of these terms, which we believe have begun to transform single-cell dynamic patterns from a purely observational and descriptive subject to more mechanistic studies. Specifically, we focus on the nature of local excitable and oscillation events, their spatial couplings leading to propagating waves and the role of active membrane. Instead of arguing for their functional importance, we prefer to consider such patterns as basic properties of dynamic systems. We discuss how knowledge of these patterns could be used to dissect the structure of cellular organization and how the network-centric view could help define cellular functions as transitions between different dynamical states. Last, we speculate on how these patterns could encode temporal and spatial information.

This article is part of the theme issue ‘Self-organization in cell biology’.

## 1. Introduction

Rhythmic dynamics of the cell cortex have been noted since light microscopy was used to observe living cells. When cells are changing shape, be it cell migration, spreading, growth or division, the changes in the cortex often happen in a pulsatile manner with different degrees of regularity. In different parts of the cell, these pulses are not always synchronized, thereby giving rise to a plethora of spatiotemporal patterns with the more coherent ones appearing to be wave-like. There are vintage examples describing compelling cortical rhythm and waves in fibroblasts [1], leucocytes [2], *Dictyostelium discoideum* [3], *Physarum polycephalum* (an enormous amoeba with neuron-like morphology) [4], eggs and embryos [5], all of which can be considered as model systems for single-cell patterns. With recent advances in visualization and biosensor development, cortical patterns can now be seen in more systems beyond these motile or large cells (see reviews in [6–10]). Examining single-cell systems offers exciting opportunities for a molecular understanding of the pattern formation process, which is typically studied in chemical systems [11] or multicellular developmental systems. Yet, the majority of cell biologists likely consider these patterns to be curious and exotic phenomena. Frequent questions include: Do these patterns only happen in specialized systems or circumstances? Are patterns functionally significant? Why do cells make patterns?

## 2. Basic theoretical frameworks of pattern formation

To start thinking about these existential problems, one first needs to appreciate that in theory patterns could spontaneously develop as the result of interactions and feedback mechanisms inherent in very simple but nonlinear dynamical systems. For temporal oscillations, the earliest model is the predator–prey model, developed in ecology almost a century ago [12,13]. The paradigm for excitable and oscillatory behaviours is the Hodgkin–Huxley (HH) model, which was developed in 1952 to describe the kinetics of action potentials in electrically excitable cells using only four ordinary differential equations [14]. The HH model and the later, more generalized, FitzHugh–Nagumo equations [15,16] are empirical models that are mathematically but not biologically tractable (the constants used in the model do not all have physical meanings). In addition, spatial patterns (propagation of the action potential from localized activation) were not addressed. However, the models illustrate how stimulation above a threshold induces a pulse of action potential followed by a refractory phase before returning to the baseline—a typical trajectory for excitable systems (see reviews in [10,17]). They also define the mathematical criteria for the existence of oscillatory dynamics [15]. These so-called limit cycle oscillations (which refers to a closed trajectory in phase space when all the states are illustrated) require negative feedback and an open system as well as an unstable steady state [18]. If the steady state is stable, the system returns to steady state after being excited, instead of continuing with another cycle.

The breakthrough for spatial pattern formation took place in the same year as the HH model, when Turing proposed his now well-known reaction–diffusion (RD) model [19]. With two partial differential equations to describe interacting reactants and their diffusions in space, a large number of spatiotemporal patterns in developmental biology could be readily obtained. Turing's model was largely unknown to the biology community until Meinhardt and Gierer proposed their pattern formation theory based on short-range activation and long-range inhibition in the 1970s [20,21]. The Meinhardt–Gierer model is considered to be largely equivalent to Turing's model (for their differences, see [22]), but their papers are more accessible to biologists [18]. These papers demonstrate that stable spatial patterns can form if the inhibitor has a longer range than the activator, temporal oscillations can form if the inhibitor has a longer life time than the activator, and a travelling wave can form if a baseline inhibitor suppresses the spontaneous activation and the activator spreads faster than the inhibitor [23].

RD models assume chemical diffusion as the main mechanism of spatial-coupling but the conceptual framework could be applied to other types of coupling, including electrical and mechanical coupling [19,24]. One can generalize this further by considering spatial pattern formation as the result of synchronization or partial synchronization of a population of coupled oscillators [25]. Here, the system is composed of phase-shifted oscillating reactions, which is mathematically described as a phase function without assuming the physical nature of the phase shifts. Coupled-oscillator models have been applied to many synchronization phenomena occurring in nature, such as the rhythmic blinking of fireflies in the dark, applause of concert audiences, and how a walking crowd caused the wobbling of London's Millennium Bridge [26]. Many of these systems are composed of discrete units of oscillatory reactions but Kuramoto showed that a coupled-oscillator model could also apply to continuous medium, such as the diffusion-coupled Belousov–Zhabotinskii reaction (the founding reaction that introduced non-equilibrium thermodynamics to chemistry and inspired models of nonlinear behaviour) [27].

Mathematically, coupled-oscillator models are more suitable to study weakly coupled (short-range) limit cycle oscillations. Thus, applying coupled-oscillator models rigorously in non-oscillating excitable systems, or in systems with non-local long-range coupling, would be non-trivial [28]. Biologically, coupled-oscillator models are attractive for developmental systems and can be argued as conceptually different from RD models [29]. In RD models, differential cell fate leading to spatial patterns originates from ‘prepatterns' in the embryos, such as a spatially distributed morphogen. Coupled-oscillator models, on the other hand, employ the temporal order to generate spatial patterns. For instance, Goodwin & Cohen proposed that phase shifts between the coupled cellular oscillators can encode size and positioning information required for developmental patterning [30]. Cooke & Zeeman also hypothesized that an endogenous oscillator of the cell can function like a clock and the propagation of the wavefront of this clock can generate periodic structures with regular spacing (the clock and wavefront model) [31,32].

Spatiotemporal patterns in single cells are typically studied with RD models and are less frequently discussed as coupled oscillators. Apart from technical difficulties in modelling, travelling waves are considered to originate more readily from excitable systems than oscillatory systems [7,33]. Intuitively, the coupled-oscillator is easily understood in discrete systems (tissue made of many single cells) as opposed to continuous systems (cortex of a single cell). The concept of the cell is deeply ingrained in our minds so it is easy to consider the cell as a discrete functional unit with autonomous properties. It does not bother us that on the time scale of the development, cells do migrate and lose their positions, and cells do divide, differentiate, and lose their identities. On the other hand, the unit of cortex is much more abstract. Whatever this unit might be, it is hard to imagine its identity with constant diffusion, flow and transport. The visual impression of the waves really challenges the spatial identity of any cortical unit. Therefore, it is not surprising that coupled oscillators are only occasionally discussed in single cells when different regions are grossly uncoupled [34,35]. However, it is important to note that a continuous and coherent system can be readily treated as weakly coupled oscillators if the diffusion terms are small compared with the reaction terms [28,36,37]. This scenario could be plausible in single cells but has not been quantitatively assessed experimentally.

Here we summarize these basic frameworks of pattern formation in order to convey a few key points. First, excitable and oscillatory dynamics and their spatial patterning can be readily explained by various models with little assumption as regards biological details. Therefore, formation of spatiotemporal patterns should be considered as ubiquitous properties of dynamical systems. This point is important because the very question of the function of the pattern is phrased within the classic framework of Darwinism, where the only source of order is through natural selection based on fitness advantages (function). If patterns are basic properties of dynamical systems in the same sense that the wave is a property of light, their existence should not be justified from a functional perspective. Rather, they reveal a tendency of self-organization in biological systems, which is likely more fundamental than any specialized function. Whether self-organization, in general, fits in the current framework of Darwinism or requires an expanded framework is a more profound open question than what we can discuss here [38]. Second, because well-ordered patterns have specific requirements, they represent a particular dynamical state of the cell. As such, they are not expected to occur all of the time when cells experience different states. Lastly, while different frameworks can potentially reproduce phenomenologically similar patterns, one needs to differentiate between empirical and mechanistic models. Because all of these models were developed long before any of the biological details were known, they provide justification for the existence of the pattern but not enough constraints to predict the biological mechanisms. There are still plenty of spaces within what is theoretically plausible, which likely corresponds to the diversity of the patterns and the uncertainty in their interpretations. Understanding how these patterns happen in any real biological setting is still challenging and will require a combination of quantitative biology and mechanistic models.

## 3. The zoo of cortical oscillation and waves

**Figure 1.**
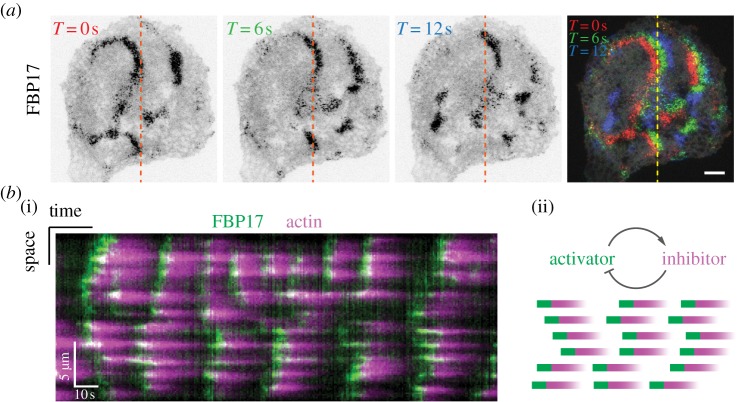
Spatiotemporal patterns in the cortex of a single cell. (*a*) Total internal reflection fluorescence image showing FBP17-EGFP waves in rat basophil leukaemia cell. The overlay image shows the time projection of waves. Scale bar, 10 µm. Inverted lookup table is used in the first three images. (*b*) (i) Kymograph of cortical waves in a cell co-transfected with FBP17-EGFP (green) and mCherry-actin (magenta). (*b*) (ii) Waves appear by phase shifts in the local oscillations.

Molecular characterizations of the cortical pattern at the single-cell level are primarily focused on actin. Actin as a cortical pattern marker is both unifying and confusing. We highlight the importance of understanding the local excitable or oscillatory dynamics prior to understanding the spatial pattern formation. When upstream factors marking the nucleation site of actin polymerization are used, it is clear that cortical waves represent clusters of discrete loci oscillating at different phases (figure 1). Sequential assembly and disassembly of the loci gives the impression of propagating waves [39–42]. This wave propagation is most clearly shown in the cortical pattern at the basal surface but the same is true for waves associated with the leading edge of the migrating cells [35,43] or neuronal branches [44]. In fact, even in the absence of high-resolution fluorescence imaging, it has been recognized that cortical waves are made up of components that are ‘stationary with respect to points outside of the cell' [45]. Such local dynamics could be superimposed with cortical flow, but the local reactions have to be dissected and understood. We, therefore, focus on the molecular understanding of the activator–inhibitor systems underlying the local reactions and spatial-coupling mechanisms that affect their propagation in space.

### (a) Activator and positive feedback mechanisms

When actin was the only marker for cortical patterns, it was difficult to agree on whether actin played an activator or inhibitor role [10,46]. Now, many upstream factors of actin have been found to display excitable or oscillatory dynamics, including various GTPases and lipid second messenger (electronic supplementary material, table S1). These additional cortical wave markers allow one to determine the effect of actin inhibition on cortical waves. It turns out that inhibition of actin leads to a longer lifetime of GTPase activation and longer oscillation periods in many cases, indicating that actin largely functions as a negative regulator in these systems [47]. Intriguingly, it is possible to observe membrane oscillations when actin is inhibited, such as the phosphatidylinositol-3,4,5-trisphosphate (PIP3) and phosphatase and tensin homologue (PTEN) oscillations in *Dictyostelium* [48,49], Cdc42 oscillations in neutrophils [50] and in yeast [51]. Although an active role of actin cannot be excluded, especially for protrusive patterns, these findings highlight the importance of self-organizing membrane-localized signalling networks as activators.

Signal amplification mechanisms such as positive feedback are frequently employed to ensure sustained oscillations [17] (for examples of alternative mechanisms such as cooperativity or ultrasensitivity, see [52]). On a molecular level, positive feedback activation of GTPases typically requires direct interaction of the guanine nucleotide exchange factor with its own product, the GTP-bound form of GTPase. Such allosteric regulation has been reported for Cdc42 [53,54] and Ras [55–58]. Alternatively, different pathways could positively feedback to each other, such as between PI3 K and Ras pathways [59,60] or between phosphatidylinositol-4,5-bisphosphate 3-kinase (PI3 K) and Rho pathways [61]. Pathway cross-talk is empirically defined without knowing the precise network structures. Lastly, a number of positive feedback loops involving actin have been proposed for stable pattern formation such as polarity. These include feedback between actin-based transport of Cdc42GTP and Cdc42GTP-stimulated actin polymerization [62,63], or between PI3 K and actin [64]. Whether the same mechanism operates in dynamic patterns is not clear.

### (b) Oscillation frequency and inhibitor identity

Not all cortical patterns are in an oscillatory state and dynamics of the local reaction vary significantly from non-recurring patterns in neutrophils [43], through recurring but irregular patterns in *Dictyostelium* [65], to more regular oscillations in *Dictyostelium* [66], mast cells [67], embryos [68] and neutrophils [50]. These dynamics likely reflect that cells exist in different states closer to either excitable or oscillatory state. An excitable state is an intermediate between a resting and oscillatory state [69]. In excitable systems, interspike time is determined by both the refractory phase and the resting phase and will lead to overestimation of the time scale of the inhibitory reaction. One way to determine the time scale of the inhibitor in non-oscillating conditions without knowing the inhibitor identity is to apply periodic stimuli and determine the resonating frequency. The resonating frequency determined this way in *Dictyostelium* cortex is around 20 s [70].

We focus on systems displaying more regular cortical oscillations because they represent experimental conditions closest to the limit cycle oscillators, which are useful conditions to reveal the underlying inhibitor kinetics. Cortical oscillations are typically on the time scale of seconds to minutes though three regimes of time scale frequently appear: a fast oscillation of around 10 s, an intermediate rhythm of 20–30 s and a slow one of between 2 and 3 min. Interestingly, mixed frequencies in the same cell were reported when cortex shape was monitored. For instance, the frequency distribution of neutrophil shape oscillations has two peaks at 8 s and 20–30 s [71,72]. In *P. polycephalum*, coexistence of two rhythms (2–3 min, 5–6 min) [73] or three rhythms (3.3 s, 24 s, 1.3 min) [74] has also been reported. Mixed frequencies, especially those in the same system, could indicate existence of cortical oscillations mediated by different activator–inhibitor networks. For instance, in mast cells, coexistence of two different rhythms of actin oscillations has been observed, with one at 30 s due to active Cdc42 pulsing and another at 100 s corresponding to active Rho pulsing [75]. If the period is different by exactly twofold, this difference could also originate from period doubling of the same oscillator [76].

Inhibitory reaction plays an important role in defining the refractory phase and setting the pace of the oscillations. The unequivocal determination of the molecular identity of the inhibitor will require reconstitution experiments that have not been reported for cortical oscillations. Acute methods are often employed to quantitatively tune oscillation frequencies in order to infer the inhibitory reactions. As mentioned above, actin appears to be a negative regulator in many of the excitable and oscillatory systems [47]. In addition, myosin II-dependent contraction is often proposed as a delayed inhibitor for oscillatory protrusion–retraction cycles, including lateral patterns at the leading edges during cell spreading [77], contracting epithelial cell–cell junctions [78] and shape oscillations observed in fibroblasts in suspensions [79]. These contractile pulses tend to have longer oscillation period (greater than 1 min) and are downstream of Rho activation [80]. However, whether myosin II is necessarily required for Rho pulses in all systems is not clear [81].

Lipid phosphatases are frequently found to be the inhibitors for basal surface patterns that are myosin II-independent [40,65]. In *Dictyostelium*, PTEN is generally modelled as an inhibitor [48,49,82,83]. In mast cells, cortical oscillations of Cdc42/F-BAR (Fer-CIP4 Homology-Bin/Amphiphysin/Rvs) proteins have typical oscillation frequencies of 30 s and these frequencies could be tuned by PI3 K activation coupled with SHIP1, a lipid phosphatase [67]. Using optogenetics to acutely elevate PI3 K levels can decrease the period to about 10 s. Conversely, addition of a chemical inhibitor of PI3 K can increase the oscillation period to up to 80 s, after which no oscillations can be observed. The limit of inhibitor lifetime in this PI3 K/SHIP1 oscillator, therefore, appears to be 10–80 s.

### (c) Spatial-coupling of the local oscillators and wave velocity

**Figure 2.**
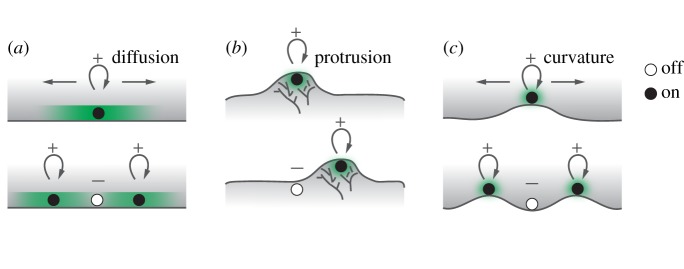
Mechanisms of spatial-coupling in cortical waves. (*a*) RD chemical waves are diffusion-coupled propagation of chemical activity. (*b*) Protrusion waves are waves driven and limited by the speed of actin polymerization, where membrane plays a passive role. (*c*) Curvature waves are powered by membrane shape undulations through the coupling with curvature-sensing chemical reactions. Black or white dots indicate the on or off state of reactions. Green shade indicates activator in waves. Grey shade marks the inside of a cell and grey branches represent actin filaments. ‘+' indicates activating signals, whereas ‘−' indicates inhibiting signals. Top to bottom are two consecutive time points.

For spatiotemporal patterns such as travelling waves, the most fundamental question is the underlying mechanisms of the spatial connectivity, which affect their propagation velocity. There are at least three types of coupling for cortical waves (figure 2) [33]. First is the pure RD chemical wave. The speed of chemical wave propagation scales with 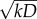, where *k* is the autocatalytic rate constant and *D* is a diffusion coefficient [84]. For cortical waves involving a membrane-localized activator, the speed is limited by membrane diffusion of the upstream regulator. The second type of wave is the membrane ‘protrusion wave' driven by actin polymerization. Velocities of these wave are limited by the rate of actin polymerization and are reduced when actin polymerization is inhibited. The third type of wave is the ‘curvature wave' driven by membrane undulations. It was predicted that active membrane coupled with proteins promoting membrane asymmetry can lead to shape changes that travel as waves [85]. Propagation velocity here will depend on the rate of curvature propagation.

Experimentally differentiating between these mechanisms requires quantitative information of the propagation velocity (electronic supplementary material, table S2) and how this velocity responds to perturbations, which has only been determined in a small number of cases. RD waves are considered the default mechanism. For actin waves observed in neuronal growth cones, wave velocity is slower with inhibited actin polymerization, which is consistent with actin-driven protrusive waves [44]. These neuronal waves, as well as dorsal protrusive waves, are slow and propagate with a rate <0.1 µm s^−1^. However, there are also exceptions. In *Dictyostelium*, the maximal velocity of the leading edge waves, which should also involve protrusion, can be as fast as 0.7 µm s^−1^ [86,87]. In addition, wave velocity could be faster when phosphatidylinositol-4,5-bisphosphate (PIP2) is depleted [88], which presumably reduces cortical actin. Basal surface waves and waves visualized at the equator plane of the cell propagate with heterogeneous speeds, whereas slow waves have a velocity around 0.01–0.2 µm s^−1^ and faster waves have a velocity around 0.5–1 µm s^−1^. Some of these faster waves appear to be actin-independent [50,89] but curvature-dependent [89].

### (d) Active membrane

While it is likely necessary to differentiate cortical waves based on their location, location alone is limited in predicting the oscillation and wave properties. A more direct parameter is perhaps the membrane curvature in the specific context of the membrane–cytoskeleton interface. Protrusive edges in *Dictyostelium* have a much less negative curvature compared with the lamellipodia in the fibroblast [86], which may explain why protrusive wave velocities are almost undiminished compared with those of the basal surface waves in *Dictyostelium* [87], but are much slower in other cell types.

Changes in membrane curvature could influence recruitment of curvature-sensing proteins, polarity of the actin assembly and its force-generation on the membrane. This chain of events, in turn, affects both the reaction kinetics and means of wave propagation. The Bin/Amphiphysin/Rvs (BAR)-domain superfamily proteins are likely candidates linking membrane curvature to the cortical pattern. F-BAR proteins (FBP17, CIP4 and Toca-1) are recruited to the basal cortical waves in mast cells [42]. This recruitment is surprising because basal surface waves are the least likely to involve membrane shape changes. An additional puzzling fact is that these F-BAR waves are faster than some other basal cortical waves or protrusive waves that also involve changes in membrane curvature. It turns out that F-BAR proteins can drive shallow inward membrane bending [89]. Changes in curvature have a non-local effect in recruiting curvature-sensing proteins and driving wave propagation. The membrane undulations have small amplitudes and cannot be supported by N-BAR proteins preferring high curvature. This is a different mechanism compared with the hypothesized involvement of curvature-sensing proteins in the self-amplification step of a protrusion wave [9]. For extensive membrane bending, actin polymerization rate is limiting and the wave spreads slowly. Experimentally, the N-BAR protein endophilin was recruited to the oscillating leading edge in fibroblasts though in the contraction phase rather than the protrusion phase [90]. Curvature is likely involved in other systems where membrane shape changes are observed but specific proteins are not identified [91,92]. Considering the versatility of BAR-domain proteins, their involvement in cortical patterns is likely to be multi-faceted. Besides shape undulation, membrane trafficking events such as endocytosis or exocytosis could also be coupled with cortical oscillations [93,94]. The active involvement of membrane is likely essential for understanding the mechanosensitivity of the cortical patterns.

## 4. Information content of the cortical patterns

The difficulty in unifying the plethora of oscillation and wave phenomena in single cells could suggest their potential to encode rich dynamic information, but their heterogeneity and potential to superimpose with each other pose great challenges in their interpretation. Because relatively little is known about their biological meanings, this section is less a summary of the progress made but more a collection of open questions about how patterns could encode information.

### (a) Probing network structure

Many current attempts to deconstruct cellular biological systems rely on dissecting molecules and their interactions. This approach is only effective if the function is encoded at the level of individual genes. Owing to the rarity of genes linked to fixed functionalities, it has been long recognized that a better proxy would be molecular networks and the interactions of these networks, both of which are flexible and dynamic [95]. However, the challenge has always been the lack of methodology to identify functional networks [96]. Oscillations are powerful readouts for understanding both the components and the topology of the biological networks [97]. Because networks need to communicate with each other, and entrainment is the most common mode of the communication, it should be fairly common for factors oscillating with the same rhythm to belong to separate feedback loops. Such information could be inferred in at least three ways: differential participation in the pattern, differential responses to perturbations and differential contributions to the pattern.

An example of differential participation in the pattern includes PIP3 and Ras, which are frequently considered to be upstream factors involved in cortical rhythms in *Dictyostelium*. However, only a subset of cells exhibiting PIP3/PTEN waves are associated with active Ras. This partial association indicates that positive feedbacks leading to localized Ras activation belong to a different network from PIP3 oscillations [48]. Similarly, cortical actin oscillations could be uncoupled from calcium oscillations, indicating that they belong to two separate networks [42]. An example of coupled oscillators that can be uncoupled by perturbations is that of how the PIP3 oscillator and actin oscillator react to an actin depolymerization drug [66]. Uncoupling of the signalling networks and actin module has led to a new model of chemotaxis that isolates gradient-sensing from motility [98]. Examples for differential contribution to the pattern are rare. We have found that while both PIP2 and PIP3 are oscillating with similar phases to FBP17 in mast cells, perturbation of PIP2 and PIP3 leads to changes in amplitude and frequency of FBP17 oscillations, respectively [67]. Mechanistically, such differential effects occur because the PIP2 network includes synaptojanin 2, the negative regulator for PIP2, as part of the incoherent feedforward loop while the PIP3 network includes SHIP1 as part of the delayed negative feedback loop. Thus, PIP2 and PIP3 function through separate networks even though they are intimately linked metabolically.

### (b) Frequency-encoded signalling and decoding

Do oscillations contain temporal information? The idea of frequency-encoded signals has been extensively reviewed for intracellular oscillations [99–102]. Yet not much is known about the information encoded by cortical oscillation frequencies. Biologically, it would be stimulation concentration [103] (this also applies to calcium oscillations [104], which are cytosolic but have a cortical origin), cell adhesion strength [105], energy state [71,106] or, at the molecular level, an enzymatic activation (PI3 K activity is frequency-coded in mast cells [67]).

**Figure 3.**
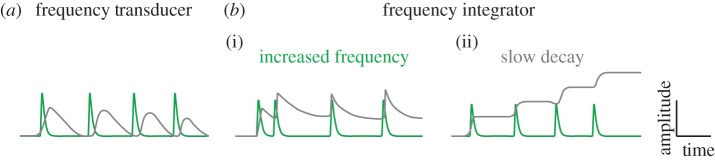
Frequency transduction and integration. Frequency information is transduced if the decay time of the signal is shorter than the oscillation period (*a*). (*b*) When frequency increases (i) or the decay time of the signal is longer than the oscillation period (ii), information will be integrated and stored as amplitude.

Whether these periodic activities could be decoded in a frequency-dependent manner is an open question. Frequency-decoding requires coupling the oscillation with a slower decay process outlasting the individual cycles of the oscillation in order to convert the frequency back to amplitude (figure 3). Such persistent responses could be considered cellular memory or a timer [107]. If the pace of cortical oscillations is in the range of 10 s to a few minutes, it is theoretically plausible that they could be integrated at the level of transcription, considering that the fastest transcriptional pulsing takes a few minutes [108,109]. If true, next it will be critical to determine whether frequency-dependent responses really count time. For instance, calcium oscillations are proposed to be frequency-decoded by build-up of dephosphorylated Nuclear factor of activated T-cells (NFATs) in the cytoplasm [110]. Yet a recent study independently controlling the duration and number of pulses using optogenetics suggests that NFAT activity depends more on the pulse duration than on the frequency. Thus, NFATs may not be a true frequency decoder or timer [111]. Intracellular signalling pathways, such as the ERK [112] and MAPK pathways [113,114], display band-pass filtering effects when challenged with periodic stimuli. These findings are consistent with frequency-decoding but whether these pathways are related to cortical oscillations is not clear. Biological time could be measured with continuous signals, such as production of an activator or decay of an inhibitor with time, but pulsed signals as timers are more tunable and adaptive [115].

### (c) Defining cellular function as state transitions

**Figure 4.**
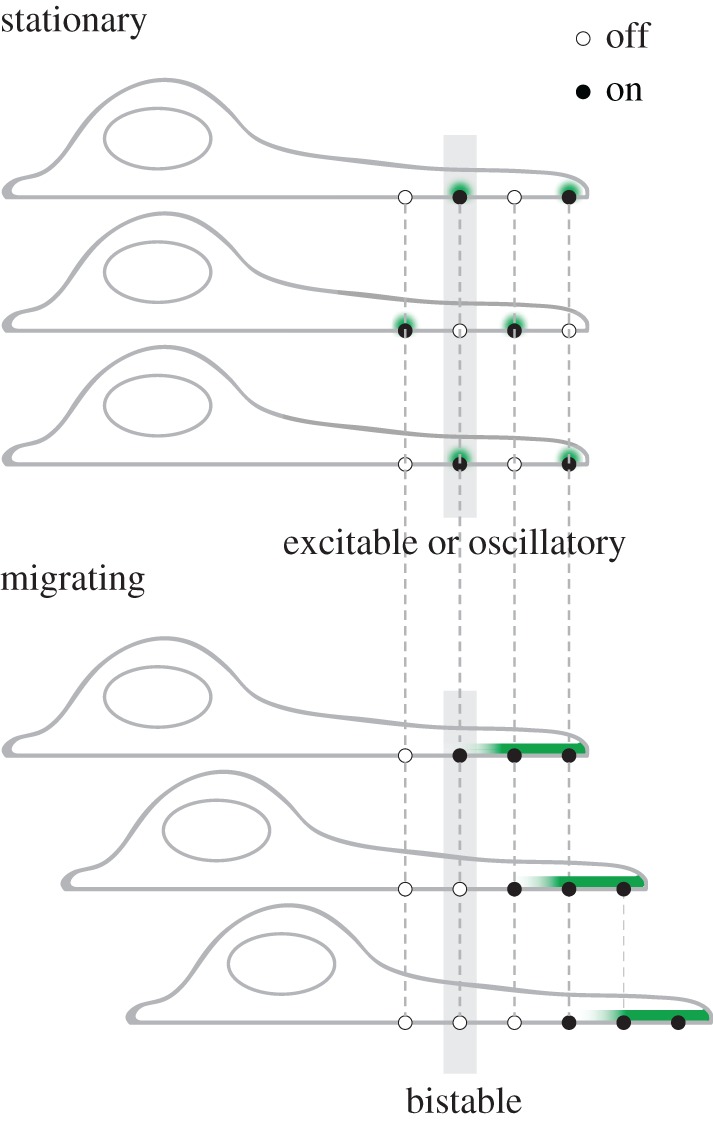
Cell migration considered as state transitions in an oscillatory or bistable regime. Top, each locus (connected by dashed lines) oscillates between the on and off state and the cell does not move. Bottom, loci at the front of the cell are on and loci at the back are off. The boundary between the bistable state shifts when the cell moves. Three time points in each state are shown. Grey shades highlight a single oscillator in each state. Green indicates the activator.

For spatial patterns, one area of particular interest is the relationship between dynamic structures such as waves, and stable spatial structures with well-recognized functionality such as polarized leading edges. Basal surface actin waves have been widely observed in migrating cells, yet not all cell migration involves formation of the waves and many stationary cells display prominent actin waves. Both coexistence of actin waves and chemotactic responses [3,43,116] and their mutual exclusion (actin waves act as a pathologic state inhibitory to migration) [35] have been reported. Thus, it may seem that actin waves are neither necessary nor sufficient for cell motility. These seemingly contradictory results could be potentially reconciled if one considers cell motility as a transition between dynamical states and how parameters of the oscillation correlate with the transition probabilities. For cells to polarize, the cortex likely switches from an excitable state (consider a fixed locus on the cortex that oscillates between on and off) to a bistable state (constantly on at the front and off at the back) (figure 4). For a polarized cell to move forward, the boundary of the bistability shifts and the cortex in the middle of the cell switches between the two steady states of the bistable state. If high amplitude oscillations imply a larger difference between the two steady states in the bistable regime, it is possible that the amplitude will be inversely correlated with the transition likelihood so that high amplitude oscillation has more difficulty transiting to migration mode. A recent study shows that changing properties of basal excitability can cause switching between random amoeba migration and persistent or oscillatory migration [88]. In particular, reduced PIP2 levels lead to faster, more frequent waves and faster migration, but these migrations are more reversible. This could represent the opposite of the above scenario if reduced PIP2 could lower PIP2-dependent cortical oscillation amplitudes [67]. As a result, the transition between states could be easier and migration is likely. At the same time switching back is also more likely, leading to reversible movements. With limited information on the determinants of many of these states, a detailed molecular model remains speculative. Yet it is clear that it is not oscillation *per se* that is functionally important or unimportant for motility, but properties of the feedback loops are linked with the dynamical states of the cell as well as the likelihood of the transition between them, all of which could be included in the same framework [117–120].

State transitions have also been discussed in the context of cell division. Formation of the stable cytokinesis furrow and travelling waves prior to furrow formation are two states depending on the level of active Rho [68,121]. Although oscillation represents an intermediate state between bistable and excitable state, it is not a compulsory intermediate. It is possible that a perturbation is so strong that the system goes directly from a resting state to a bistable state without going through an excitable or oscillatory intermediate. When cells are not oscillating, or do not go through the oscillating state, it does not mean that the network defined by oscillations does not exist. It only means that cells narrowly miss the set of parameters that support limit cycles.

### (d) Spatial singularity and size information

Do waves contain spatial information? In a now classic review on the role of waves in a developmental context, Goodwin classified waves into three categories based on the information they provide: the S-wave for synchronization, the P-wave for positional information and the R-wave for size regulation [30]. With the exception of situations where the phase differences could be ignored and waves could be used to synchronize cellular activity such as cell cycle [122], it is unlikely that S-wave applies to entities such as single cells. Could cortical waves encode position or size information? An emerging theme relating to position information is that cortical waves could be used to ensure singularity in spatial events. In embryos, fusion of the sperm and egg plasma membranes leads to propagation of fast waves of membrane depolarization followed by calcium waves. The function of the activation wave is thought to ensure that only one sperm fertilizes a given egg. The refractory phase of the activation wave effectively blocks polyspermy [123]. In yeast, formation of a polarity site is accompanied with travelling waves of Cdc42. Since multiple buds could form either spontaneously [124] or when positive feedback is strengthened [62], it was hypothesized that negative feedback could help ensure the robust formation of a single bud despite the wide variability of activator level [51]. The precise role of negative feedback (i.e. travelling waves) remains to be experimentally evaluated by uncoupling the activation level and wave propagation range. In *Escherichia coli*, pole-to-pole oscillation of Min protein waves is thought to prevent assembly of cell division machinery anywhere but at the centre of the cell [125]. In longer bacteria, Min protein waves become multi-segmented. In eukaryotic cells, occurrence of Cdc42 waves during mitosis also prevents formation of multiple furrows [75]. In all of these examples, the refractory phase sets the length scale and provides negative signals to ensure spatial singularity within this length scale, which can be considered as negative selection. Mitotic cortical Cdc42 waves could also have some positive positioning function [75]. Intriguingly, a recent study using fast atomic force microscopy imaging found that bacterial division occurs at the wave troughs on the undulating cell surface, formation of which is the earliest events for division site selection [126]. Although the molecular marker is not known, this points to a potential general role of membrane waves for positioning cell events.

**Figure 5.**
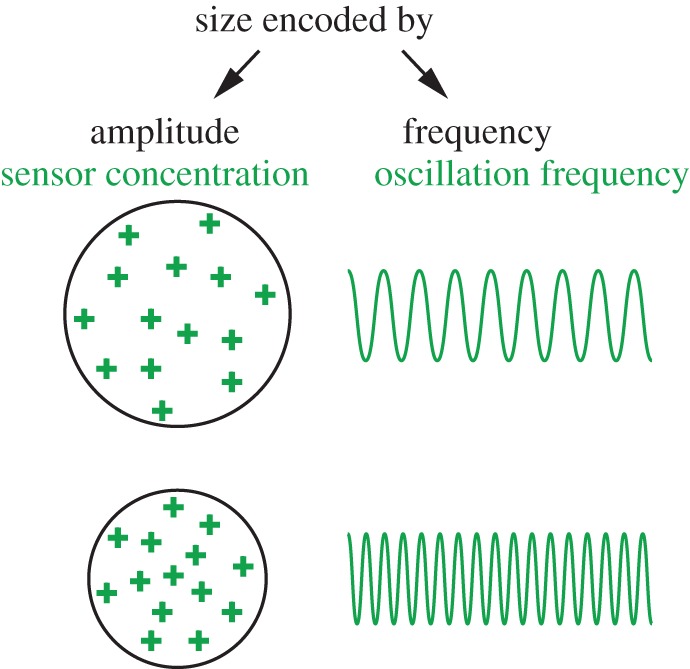
Schematic of how size information could be amplitude- or frequency-coded. Area is inversely proportional to sensor concentration (amplitude-encoded), which could be converted into oscillation frequencies (frequency-encoded). If sensor amount is size-independent, larger cells have lower concentration of the sensor (in amplitude mode) or lower oscillation frequency (in frequency mode), while smaller cells have higher concentration or frequency. Thus, concentration or frequency could encode cell sizes.

How a cell determines its size or length is one of the major unsolved questions in cell biology [24,127]. Whether a cell even knows its size remains heavily debated [128,129]. Theoretically, it would be easy to turn an oscillator into a measuring device if one could tune the oscillation frequency without changing wave propagation speed [24], in which case size could be frequency-encoded. However, when cortical waves were studied in giant cells of *Dictyostelium*, their wavelength was found to be an intrinsic property that does not vary with cell size [65]. In *E. coli* oscillation periods of Min waves appear to change with the lengths of the bacteria, with faster oscillations in shorter cells [130–132]. If cell division is inhibited, the period of MinD waves doubles [125,133]. In *P. polycephalum*, cell size oscillation could be measured as an oscillation in cell thickness, the period of which scales with the thickness of the cell [134]. At the molecular level, size-sensing is poorly defined. In budding yeast, Whi5 has been reported as a size sensor. It has cell-size-independent expression, so smaller cells have proportionally higher concentration. Growth-induced dilution of Whi5 can, therefore, sense size and gate G1/S entry [135]. This is an example where cell size is amplitude-encoded, and cell sizes could equally be frequency-encoded (figure 5). For instance, for cortical oscillations whose frequencies are proportional to PIP3 concentration, if the total amount of PIP3 is constant for both large and small cells, the oscillation frequency could be used to encode size information [75].

## 5. Concluding remarks

There is a natural tendency to speak of the function of oscillations or waves. The search for such a function will continue because an absence of functionality cannot be proven [136]. In non-biological systems such as electronics, all feedback loops potentially have the capacity to oscillate, and some of the oscillations are considered undesirable side effects and called parasitic oscillations. In complex cellular systems where the criteria of optimal performance are still evolving [137], debating the function of the pattern is likely unproductive. After all, even though networks are better proxies for function than individual genes, they are still the building blocks of the cell. Studies of chemotaxis are great examples where information from the excitable and oscillatory networks can be integrated to refine and expand our definitions of cellular functionality. Instead of being a single function, chemotaxis is now an encompassing, modular and intelligent system that includes direction and gradient-sensing, polarity establishment, cell motility, cell-turning and more. We anticipate that a better mechanistic understanding of the feedback networks and quantitative control of these signals will lead to a better understanding of cell behaviour not only as the results of genes and their interactions, but as dynamical networks and their integrations.

## Supplementary Material

Supplemental tables

## Data accessibility

This article has no additional data.
