# Supplementary material for: Rhythmicity and waves in the cortex of single cells
Source: Philos Trans R Soc Lond B Biol Sci. 2018 Apr 9;373(1747):20170116. doi: 10.1098/rstb.2017.0116 (PMC5904302; doi:10.1098/rstb.2017.0116)
Supplement: Supplemental tables [file rstb20170116supp1.pdf]

Supplemental Tables

Cell type	Upstream factor	Require actomyosin contractility?	Location	Membrane deformation?	Periodicity
**Slime mold**					
Dictyostelium	Ras, Rac, PIP3/PI3K [1-4];	No	Basal surface	No [5-7]	10 sec [4];
		No	Leading edge	Yes [8]	200 sec[4]
	PI3K, PTEN [8,9]; Ras [4]	No	Equator	n.d.	~200 sec [9]
P. polycephalum		Yes		Likely yes.	50-90 sec [10]
**Leukocyte**					
Neutrophils (HL-60)	Rac, Hem1 (SCAR/WAVE) [11], PI3K, TORC2, PLA2 [12]	No	Basal surface	n.d.	10 sec [12]
Neutrophils (PLB-985)	Cdc42 [13]		Leading edge	Likely yes.	~25 sec [13]
Mast cells (RBL-2H3)	Cdc42 [14]; PIP2, PIP3, PI3K, SHIP1 [15]	No	Basal surface	Yes. F-BAR FBP17 [14] and CIP4	~30 sec [14]
**Eggs and embryos**					
Xenopus and starfish	Rho [16]	No	Basal surface	n.d.	80-120 sec [16]
Mouse embryo		Yes	Equator	Curvature wave [17]	90 sec [17]
**Fibroblast**					
MEF, HeLa, BHK21,	PI3K, Rac [18]	No;	Basal surface	Yes[18]	130-200 sec [19]
3T3		Yes [20]	Leading edge	Yes. N-BAR Nadrin 2, Amphiphysin 1 [20]	~200 sec [20]
**Epithelial**					
Drosophila follicle cells	Rho [21]	Yes	Equator	Likely yes.	300 sec [21]
Drosophila dorsal closure		Yes	Equator	Likely yes.	230 sec [22]
**Neurons**					
Neurons	Cdc42 [23,24], Rap1B, Rac [24]	No [24]	Leading edge	Likely yes.	>30 min [23-26]

Supplemental Table 1: Summary of the properties of periodic travelling waves in the single cells.

Cell type	Basal surface waves	Equator waves	Leading edge waves	Other waves
Dictyostelium	0.01-0.2 [1-3,5-7,27-29]; 0.21-0.76 [4,30,31]		0.3-0.7 [32]	
P. polycephalum				1-20 [33] (amplitude wave)
Neutrophils	1.1 [13]		0.08 [11]	
Mast cells	0.5-1 [14]			
Eggs and embryos	0.22 [16]	0.8**[17]		
Fibroblasts	0.027 [18], 0.2 [34] up to 1 [28]		0.1 [35]; 0.07 [36]	0.012-0.038 [37]; 0.12 [38] (apical waves)
Neurons			0.03 [23]	

Supplemental Table 2: Summary of actin wave velocity (unit: µm/sec).

References

1. Asano, Y., Nagasaki, A. & Uyeda, T. Q. P. 2008 Correlated waves of actin filaments and PIP3 in Dictyostelium cells. *Cell Motil Cytoskeleton* **65**, 923–934. (doi:10.1002/cm.20314)

2. Gerisch, G., Schroth-Diez, B., Müller-Taubenberger, A. & Ecke, M. 2012 PIP3 Waves and PTEN Dynamics in the Emergence of Cell Polarity. *Biophys J* **103**, 1170–1178. (doi:10.1016/j.bpj.2012.08.004)

3. Gerhardt, M., Ecke, M., Walz, M., Stengl, A., Beta, C. & Gerisch, G. 2014 Actin and PIP3 waves in giant cells reveal the inherent length scale of an excited state. *J Cell Sci* **127**, 4507–4517. (doi:10.1242/jcs.156000)

4. Huang, C.-H., Tang, M., Shi, C., Iglesias, P. A. & Devreotes, P. N. 2013 An excitable signal integrator couples to an idling cytoskeletal oscillator to drive cell migration. *Nat Cell Biol* **15**, 1307–1316. (doi:10.1038/ncb2859)

5. Schroth-Diez, B., Gerwig, S., Ecke, M., Hegerl, R., Diez, S. & Gerisch, G. 2009 Propagating waves separate two states of actin organization in living cells. *HFSP J* **3**, 412–427. (doi:10.2976/1.3239407)

6. Gerisch, G., Bretschneider, T., Müller-Taubenberger, A., Simmeth, E., Ecke, M., Diez, S. & Anderson, K. 2004 Mobile actin clusters and traveling waves in cells recovering from actin depolymerization. *Biophys J* **87**, 3493–3503. (doi:10.1529/biophysj.104.047589)

7. Bretschneider, T., Diez, S., Anderson, K., Heuser, J., Clarke, M., Müller-Taubenberger, A., Köhler, J. & Gerisch, G. 2004 Dynamic actin patterns and Arp2/3 assembly at the substrate-attached surface of motile cells. *Curr Biol* **14**, 1–10.

8. Taniguchi, D., Ishihara, S., Oonuki, T., Honda-Kitahara, M., Kaneko, K. & Sawai, S. 2013 Phase geometries of two-dimensional excitable waves govern self-organized morphodynamics of amoeboid cells. *Proceedings of the National Academy of Sciences* **110**, 5016–5021. (doi:10.1073/pnas.1218025110)

9. Arai, Y., Shibata, T., Matsuoka, S., Sato, M. J., Yanagida, T. & Ueda, M. 2010 Self-organization of the phosphatidylinositol lipids signaling system for random cell migration. *Proceedings of the National Academy of Sciences* **107**, 12399–12404. (doi:10.1073/pnas.0908278107)

10. Takagi, S. & Ueda, T. 2008 Emergence and transitions of dynamic patterns of thickness oscillation of the plasmodium of the true slime mold Physarum polycephalum. *Physica D: Nonlinear Phenomena* **237**, 420–427. (doi:10.1016/j.physd.2007.09.012)

11. Weiner, O. D., Marganski, W. A., Wu, L. F., Altschuler, S. J. & Kirschner, M. W. 2007 An Actin-Based Wave Generator Organizes Cell Motility. *PLoS Biol* **5**, e221. (doi:10.1371/journal.pbio.0050221)

12. Hoeller, O., Toettcher, J. E., Cai, H., Sun, Y., Huang, C.-H., Freyre, M., Zhao, M., Devreotes, P. N. & Weiner, O. D. 2016 Gβ Regulates Coupling between Actin Oscillators for Cell Polarity and Directional Migration. *PLoS Biol* **14**, e1002381. (doi:10.1371/journal.pbio.1002381.s029)

13. Yang, H. W., Collins, S. R. & Meyer, T. 2016 Locally excitable Cdc42 signals steer cells during chemotaxis. *Nat Cell Biol* **18**, 191–201. (doi:10.1038/ncb3292)

14. Wu, M., Wu, X. & De Camilli, P. 2013 Calcium oscillations-coupled conversion of actin travelling waves to standing oscillations. *Proceedings of the National Academy of Sciences* **110**, 1339–1344. (doi:10.1073/pnas.1221538110)

15. Xiong, D., Xiao, S., Guo, S., Lin, Q., Nakatsu, F. & Wu, M. 2016 Frequency and amplitude control of cortical oscillations by phosphoinositide waves. *Nat Chem Biol* **12**, 159–166. (doi:10.1038/nchembio.2000)

16. Bement, W. M. et al. 2015 Activator–inhibitor coupling between Rho signalling and actin assembly makes the cell cortex an excitable medium. *Nat Cell Biol* **17**, 1471–1483. (doi:10.1038/ncb3251)

17. Maître, J.-L., Niwayama, R., Turlier, H., Nédélec, F. & Hiiragi, T. 2015 Pulsatile cell-autonomous

contractility drives compaction in the mouse embryo. *Nat Cell Biol* **17**, 849–855. (doi:10.1038/ncb3185)

18. Case, L. B. & Waterman, C. M. 2011 Adhesive F-actin Waves: A Novel Integrin-Mediated Adhesion Complex Coupled to Ventral Actin Polymerization. *PLoS ONE* **6**, e26631. (doi:10.1371/journal.pone.0026631.g007)

19. Ryan, G. L., Watanabe, N. & Vavylonis, D. 2012 A review of models of fluctuating protrusion and retraction patterns at the leading edge of motile cells. *Cytoskeleton* **69**, 195–206. (doi:10.1002/cm.21017)

20. Galic, M., Jeong, S., Tsai, F.-C., Joubert, L.-M., Wu, Y. I., Hahn, K. M., Cui, Y. & Meyer, T. 2012 External push and internal pull forces recruit curvature-sensing N-BAR domain proteins to the plasma membrane. *Nat Cell Biol* **14**, 874–881. (doi:10.1038/ncb2533)

21. He, L., Wang, X., Tang, H. L. & Montell, D. J. 2010 Tissue elongation requires oscillating contractions of a basal actomyosin network. *Nat Cell Biol* **12**, 1133–1142. (doi:10.1038/ncb2124)

22. Solon, J., Kaya-Copur, A., Colombelli, J. & Brunner, D. 2009 Pulsed Forces Timed by a Ratchet-like Mechanism Drive Directed Tissue Movement during Dorsal Closure. *Cell* **137**, 1331–1342. (doi:10.1016/j.cell.2009.03.050)

23. Winans, A. M., Collins, S. R. & Meyer, T. 2016 Waves of actin and microtubule polymerization drive microtubule-based transport and neurite growth before single axon formation. *eLife* (doi:10.7554/eLife.12387.001)

24. Flynn, K. C., Pak, C. W., Shaw, A. E., Bradke, F. & Bamburg, J. R. 2009 Growth cone-like waves transport actin and promote axonogenesis and neurite branching. *Dev Neurobiol* **69**, 761–779. (doi:10.1002/dneu.20734)

25. Katsuno, H., Toriyama, M., Hosokawa, Y., Mizuno, K., Ikeda, K., Sakumura, Y. & Inagaki, N. 2015 Actin Migration Driven by Directional Assembly and Disassembly of Membrane-Anchored Actin Filaments. *CellReports*, 1–14. (doi:10.1016/j.celrep.2015.06.048)

26. Ruthel, G. & Banker, G. 1999 Role of moving growth cone-like 'wave' structures in the outgrowth of cultured hippocampal axons and dendrites. *Journal of Neurobiology* **39**, 97–106.

27. Bretschneider, T., Anderson, K., Ecke, M., Müller-Taubenberger, A., Schroth-Diez, B., Ishikawa-Ankerhold, H. C. & Gerisch, G. 2009 The three-dimensional dynamics of actin waves, a model of cytoskeletal self-organization. *Biophys J* **96**, 2888–2900. (doi:10.1016/j.bpj.2008.12.3942)

28. Vicker, M. G. 2002 Eukaryotic Cell Locomotion Depends on the Propagation of Self-Organized Reaction–Diffusion Waves and Oscillations of Actin Filament Assembly. *Exp Cell Res* **275**, 54–66. (doi:10.1006/excr.2001.5466)

29. Vicker, M. G. 2002 F-actin assembly in Dictyostelium cell locomotion and shape oscillations propagates as a self-organized reaction-diffusion wave. *FEBS Lett* **510**, 5–9.

30. Vicker, M. G. 2000 Reaction-diffusion waves of actin filament polymerization/depolymerization in Dictyostelium pseudopodium extension and cell locomotion. *Biophys. Chem.* **84**, 87–98.

31. Vicker, M. G., Xiang, W., Plath, P. J. & Wosniok, W. 1997 Pseudopodium extension and amoeboid locomotion in Dictyostelium discoideum: possible autowave behaviour of F-actin. *Physica D: Nonlinear Phenomena* **101**, 317–332.

32. Lange, M., Prassler, J., Ecke, M., Müller-Taubenberger, A. & Gerisch, G. 2016 Local Ras activation, PTEN pattern, and global actin flow in the chemotactic responses of oversized cells. *J Cell Sci* **129**, 3462–3472. (doi:10.1242/jcs.191148)

33. Alim, K., Andrew, N., Pringle, A. & Brenner, M. P. 2017 Mechanism of signal propagation in Physarum polycephalum. *Proceedings of the National Academy of Sciences* **Vol 1**, 201618114. (doi:10.1073/pnas.1618114114)

34. Mitsushima, M., Aoki, K., Ebisuya, M., Matsumura, S., Yamamoto, T., Matsuda, M., Toyoshima, F. & Nishida, E. 2010 Revolving movement of a dynamic cluster of actin filaments during

mitosis. *J Cell Biol* **191**, 453–462. (doi:10.1083/jcb.201007136)

35. Döbereiner, H.-G., Dubin-Thaler, B. J., Hofman, J. M., Xenias, H. S., Sims, T. N., Giannone, G., Dustin, M. L., Wiggins, C. H. & Sheetz, M. P. 2006 Lateral membrane waves constitute a universal dynamic pattern of motile cells. *Phys. Rev. Lett.* **97**, 038102.

36. Giannone, G., Dubin-Thaler, B. J., Döbereiner, H.-G., Kieffer, N., Bresnick, A. R. & Sheetz, M. P. 2004 Periodic lamellipodial contractions correlate with rearward actin waves. *Cell* **116**, 431–443.

37. Peleg, B., Disanza, A., Scita, G. & Gov, N. 2011 Propagating Cell-Membrane Waves Driven by Curved Activators of Actin Polymerization. *PLoS ONE* **6**, e18635. (doi:10.1371/journal.pone.0018635.t001)

38. Bernitt, E., Koh, C. G., Gov, N. & Döbereiner, H.-G. 2015 Dynamics of Actin Waves on Patterned Substrates: A Quantitative Analysis of Circular Dorsal Ruffles. *PLoS ONE* **10**, e0115857. (doi:10.1371/journal.pone.0115857.s019)
